# PP12 Government Agency For Health Technology Assessment In Mendoza, Argentina: Experience And Path

**DOI:** 10.1017/S0266462325102328

**Published:** 2025-12-29

**Authors:** Jorgelina Alvarez, Cristina Gatica, Rodolfo Montero

## Abstract

**Introduction:**

There are big challenges in Latin America and Argentina in terms of access to health. There is also consensus on the importance of managing access, coverage of health technology assessment (HTA), and the achievement of more efficient health expenditure. Our aim was to describe the creation and implementation of an Argentinian government HTA agency.

**Methods:**

A local HTA agency project was implemented in Mendoza, Argentina based on: (i) the experience of an active local government HTA committee; and (ii) local and national consultation with decision-makers, health funders, and providers. An institutionalization plan for HTA was implemented based on a set of interventions that included legal framework, resources, and institutional agreements. It was then submitted for legislative and citizen debate. Following achievement of the legal framework, HTA agency work began.

**Results:**

The Agencia de Evaluación de Tecnologías Sanitarias de Mendoza (AETS Mendoza) was created by Law 9547 (1 July 2024), regulatory decree (Dec.1777/24). HTA good practice recommendations were achieved: autonomous body with legal and budgetary independence and functions to evaluate and issue recommendations on the value of technologies in key aspects of their life cycle (efficacy, clinical relevance, cost effectiveness, budgetary impact, equity). A multidisciplinary technical team was constituted. Recommendations that emerged from the evaluations are mandatory for public sector and provincial social security funders. The HTA reports are planned through annual prioritization and published on the official website.

**Conclusions:**

HTA agencies are regulatory bodies that assess the value of health technologies and give local health systems responses to innovation, incremental spending, and equity. AETS Mendoza is the first government HTA agency in Argentina. This experience is innovative given the fragmentation and complexity of the Argentine health system. This model could be adopted by other health systems.

